# Seroepidemiology of Camel Brucellosis in and around Dire Dawa, Eastern Ethiopia

**DOI:** 10.1155/2022/6624293

**Published:** 2022-03-16

**Authors:** Hika Waktole, Mohammed Aden, Hagos Ashenafi

**Affiliations:** ^1^Addis Ababa University, College of Veterinary Medicine and Agriculture, Department of Microbiology, Immunology and Veterinary Public Health, Bishoftu, Ethiopia; ^2^Addis Ababa University, Aklilu Lemma Institute of Pathobiology, Animal Health and Zoonotic Diseases Research Unit, Addis Ababa, Ethiopia

## Abstract

Brucellosis is an infectious disease in domestic and wild animals with serious zoonotic and economic implication in humans, being more severe in developing countries. The disease is highly prevalent in cattle, camels, and small ruminants in pastoral and agro-pastoral areas in Africa. Here we have investigated the seroepidemiology of camel brucellosis in and around Dire Dawa, eastern Ethiopia, using a cross-sectional study design to determine the seroprevalence of the disease and to identify risk factors that would facilitate the transmission of zoonotic diseases to humans. This study involved testing 350 serum samples from camels and interviewing 120 livestock owners. The modified Rose Bengal plate test (mRBPT) and the complement fixation test (CFT) were used as screening and confirmatory tests, respectively. The overall sero-prevalence of camel brucellosis was found to be 8.3% and 2% using mRBPT and CFT tests, respectively. Among the risk factors assessed, only abortion and body condition disclosed a statistically significant difference (*p* < 0.05) with regard to the seropositivity of camel brucellosis. Camel brucellosis is prevalent in eastern Ethiopia and there is a need to execute well-organized disease control and prevention programs and exercise public health education to scale up awareness of the community towards the disease.

## 1. Introduction

Camels are a subset of the huge livestock resources in Ethiopia, with the population estimated to be 1.2 million. This number ranks the country third in Africa after Somalia and Sudan and fourth in the world [1]. The arid and semi-arid areas of Ethiopia constitute more than 60% of the total area suitable for camel production, ensuring the livelihood of the pastoral communities [2].

Brucellosis is a disease of high economic and public health importance, having worldwide distribution [3]. The magnitude of this disease in developing countries is more severe due to lack of appropriate control measures [4]. Brucellosis is a widely spread disease of camels in eastern African countries such as Ethiopia, Eritrea, Somalia, and Sudan. In Ethiopia, few field surveys have been carried out to determine the magnitude of camel brucellosis in pastoral areas. Brucellosis is an endemic and the disease is highly prevalent in cattle, camels, and small ruminants in pastoral and agro-pastoral areas [5].

Brucellosis is known for its zoonotic importance where it stands in the second position, and annually about 500,000 human cases are reported [6]. The disease affects almost all domestic species, and there is a high chance of cross-transmission among cattle, sheep, goats, camels, and other species [7]. Brucellosis is characterized by huge wastage in productivity in terms of abortions (late-term), weak calves, stillbirths, and infertility accompanied by placentitis, epididymitis, and orchitis.

Camel brucellosis can be caused by *Brucella abortus, B. melitensis*, and *B. ovis* [8]. However, several studies revealed that *B. abortus* and *B. melitensis* are most frequently isolated from milk, aborted fetuses, and vaginal swabs of infected camels [9–13]. Camels are not known to be the primary hosts of *Brucella*. However, *Brucella* species that occur in camels are linked and associated with those species affecting other animals [14]. As a result, the prevalence of camel brucellosis and the causative *Brucella* species depends upon the infection rate and circulating species of primary hosts being in contact with them.

Despite the endemic nature of brucellosis in many developing countries, the disease remains under-investigated in camels in Ethiopia. In light of the above, the present study was initiated and designed to determine the seroprevalence of camel brucellosis in and around Dire Dawa, eastern Ethiopia. Therefore, the study aims to investigate associated risk factors for brucellosis in camels and to assess the awareness level of the Dire Dawa pastoral community about zoonotic brucellosis.

## 2. Materials and Methods

### 2.1. Study Area

The present study was carried out from November 2018 to April 2019 in and around Dire Dawa. Dire Dawa is located at a distance of 515 kilometers from Addis Ababa in the eastern part of Ethiopia. The study area lies between 1000 and 2000 meter above sea level, between 09°28′N and 09°49′N latitude and 41°38′ to 42°19′E. The study area lies within a valley and is surrounded by the eastern mountains of eastern Hararghe and is at the verge of the semi-desert of the Somali region. The areas has a bi-modal rainfall pattern with the highest rainfall in July and August, with an average annual rainfall that varies from 700 to 900 mm. The mean annual temperature varies from 20°C to 30°C. The hottest month of the year ranges from 28.1 °C to 34.6 °C [15].

### 2.2. Study Design and Sampling Method

A cross-sectional study design was employed to study the seroepidemiology of camel brucellosis in and around Dire Dawa, eastern Ethiopia, and to identify risk factors associated with seropositivity. Information on each animal such as its location, age, sex, physiological status, reproductive history, and co-existence with other ruminants, was properly collected. Camels above 6 months of age and with no history of vaccination against brucellosis were considered in the study.

### 2.3. Sampling Method

A multistage sampling technique was used in the present study of seroprevalence survey of camel brucellosis. Accordingly, the Peasant Associations (PAs) were regarded as the primary units, the herds as the secondary units, and the individual animals as the tertiary units. A total of 50 camel herds in 4 PAs out of the 8 PAs found in Dire Dawa were sampled during the study in alignment with the camel population of each PA. In order to determine the desired sample size of camels, the lack of previous data on the prevalence of camel brucellosis in Dire Dawa was considered. Hence, the sample size was calculated, taking into account, 50% expected prevalence of Dire Dawa and 95% Confidence Intervals (CI) at 5% desired accuracy [16]. Therefore, 350 camels were sampled. Sampling was proportionally distributed based on the total camel population in the study PAs (Table 1). Moreover, a total of 120 willingly selected pastoralists were included in the study for the purpose of a questionnaire survey. Sera were collected from the camels, and then, questionnaires were administered to each randomly selected livestock owner.

### 2.4. Blood Sample Collection

By using plain vacutainer tubes, blood samples were collected from the jugular vein of camels. The collected blood samples were allowed to clot at room temperature. Then, serum was separated and decanted to screw tight 1.5 mL eppendrof tubes. Collected sera were stored at -20 °C until the laboratory tests were performed using the modified Rose Bengal plate test (mRBPT) and complement fixation test (CFT).

### 2.5. Serological Tests

#### 2.5.1. Modified Rose Bengal Plate Test (mRBPT)

The modified Rose Bengal plate test (mRBPT) was conducted in the Dire Dawa Regional Veterinary Diagnostic and Research Laboratory in order to screen positive samples using the mRBPT antigen (Institue Pourquier 325, rue de la galèra 34097 Montpellier cedex 5, France). Positive sera for mRBPT were then retested using the complement fixation test at the National Veterinary Institute (NVI) in Debre Zeit for confirmation. Samples were considered positive for brucellosis if they will be positive for mRBPT and CFT on a serial reading basis.

For the mRBPT, the sera and antigen were taken out of the refrigerator and left at room temperature for at least 30 minutes. Then, 75 *μ*l of test sera was dispensed on each of the 12 circles of the plate. The antigen bottle is gently shaked and a drop of mRBPT antigen (25 *μ*l) is place alongside the serum. The antigen and serum were mixed thoroughly using an applicator stick, and the plate was rocked manually for about 4 minutes. Finally, agglutination reactions was read in a good light source or using a magnifying glass when micro-agglutinations felt suspicion [17]. Reactions were categorized as 0, +, ++, and +++, where 0 = no agglutination, + = barely perceptible agglutination (using magnifying glass), ++ = fine agglutination (some clearing), and +++ = clumping, definite clearing. Those samples identified with no agglutination (0) were regarded as negative, while those with +, ++, and +++ were regarded as positive [18].

#### 2.5.2. Complement Fixation Test (CFT)

A 2% suspension of camel red blood cells was prepared before being used in the test. The preparation of the reagents and the CFT procedure was conducted according to the CFT at the National Veterinary Institute (NVI), Ethiopia, A serum giving 75% fixation of the complement at a dilution of 1 : 5 and above was taken as positive [19].

### 2.6. Data Analysis

All data obtained were entered into Microsoft Excel 2010 and coded data were stored and finally transferred to STATA® 13.0 for statistical analysis. Analyzing the effects of different potential risk factors on the seroprevalence of brucellosis at both the individual and group level was performed by logistic regression. The sero-prevalence was calculated by dividing the number of CFT and RBPT positive animals by the total number of animals tested. The Chi-square test was utilized to measure the association between the sero-prevalence and categorical variables. For statistical inference, a *p* value less than 0.05 was considered statistically significant.

## 3. Results

### 3.1. Seroprevalence of Camel Brucellosis

In the present study, the overall seroprevalence of camel brucellosis in Dire Dawa, eastern Ethiopia was found to be 8.3% (29/350) using the mRBPT and 2% (7/350) by the CFT tests. Moreover, the seroprevalence of camel brucellosis using mRBPT and CFT tests for the various explanatory variables is depicted in Table 2.

The seroprevalence of camel brucellosis in three age groups is presented in Table 2. None of the camels less than 3 years of age were seropositive. This study revealed a higher seroprevalence of camels with brucellosis among females (2.23%) than their male counterparts (1.5%). However, there was no statistically significant difference (*p* > 0.05) between the seroprevalence of camel brucellosis and sex. Camels having poor body condition scores had a higher seroprevalence of brucellosis (6.7%) compared with animals in the good body condition category (1.03%).

Similar to mRBPT, none of the camels less than 3 years of age were seropositive for CFT. Higher sero-prevalence camel of brucellosis was encountered among females (2.4%) than their male counterparts (1.4%). There was no statistically significant difference (*p* > 0.05) between the CFT-based sero-prevalence of camel brucellosis with that of sex and age. On the other hand, there was a statistically significant difference (*p* < 0.05) between the sero-prevalence of camel brucellosis and body condition score (Table 3).

The seroprevalence of camel brucellosis on the basis of herd size was found to be 3.8%, 1.7%, and 1.01% in large, medium, and small herd sizes, respectively. Camels with a history of previous abortion had a higher seroprevalence of brucellosis (12.5%) as opposed to nonaborted animals (1.04%). This was found to reveal a statistically significant difference (*p* < 0.05) (Table 4).

There was no statistically significant difference (*p* > 0.05) between the seroprevalence of camel brucellosis and contact with other ruminant animals. There was variation in the seroprevalence of camel brucellosis among different parity levels. It was disclosed that camels with no parturition had 0% brucellosis seroprevalence. Moreover, camels with one, two, and more than 3 parity levels had 1.5%, 5.5%, and 2.85% seroprevalence of brucellosis, respectively. The difference in the sero-prevalence of camel brucellosis and parity levels was found to be statistically not significant (*p* < 0.05) (Table 4).

## 4. Discussion

Brucellosis is a disease of high economic and public health importance and has a worldwide distribution. The magnitude of this disease in developing countries is more severe due to lack of appropriate control measures [4]. The epidemiology of brucellosis in cattle and small ruminants in different geographical locations has been investigated extensively. However, research on the epidemiology of camel brucellosis is very scarce [20]. Though brucellosis is widespread and rampant in Eastern African countries, in Ethiopia few field surveys have been carried out to determine the magnitude of camel brucellosis in pastoral areas. Brucellosis is endemic and the disease is highly prevalent in cattle, camels, and small ruminants in pastoral and agro-pastoral areas [5]. Despite the endemic nature of brucellosis in many developing countries, the disease remains underdiagnosed and under-reported [7].

The present study revealed a 2% overall seroprevalence of camel brucellosis, which was found to be in agreement with the previous reports that disclosed 2.43% in Jijiga and Babile districts of eastern Ethiopia [21], 1.9% in Somalia [22], and 1.8% from Borena lowland, southern Ethiopia [23]. However, relatively higher seroprevalence of camel brucellosis has been recorded in Sudan 30.5% [24], 23.8% in Darfur, western Sudan [14], 19.4% in Jordan [25], 7.3% in Egypt [26], 4.3–8.6% in Saudi Arabia [9], and 3.1% in Somalia [27]. The observed variations in the seroprevalence of camel brucellosis in different countries might be due to differences in management and husbandry practices, the virulence of the organism, coverage and quality of veterinary services, degree of awareness, the extent of susceptibility of the animals, and unrestricted movement of among pastoralists people [24].

The low seroprevalence of camel brucellosis observed in the present study might be due to the low density of camel population kept on widely extended grazing land and the presence of many watering points in the river path of the valleys, which reduce the concentration and close contact of camels. Moreover, the good practice of herders' evidenced by the timely culling of aborted and nonconceiving females from the herds might have contributed to the situation [28].

This study also showed a higher seroprevalence of brucellosis in females (2.4%) than in males (1.4%). The same trend of higher seroprevalence of camel brucellosis was disclosed in females (1.9%) as opposed to males (1.3%). The influence of sex in the prevalence of brucellosis has been studied in domestic and wild animals [29]. In camels, females are more susceptible to brucellosis than males. This relatively higher susceptibility of female camels could be due to the fact that they have more physiological stress than males [30]. Male animals are less susceptible to brucella infection due to the absence of erythritol sugar, which is found in the uterus [31]. Also female camels are kept longer in herds for breeding purposes than male camels, which are fattened and sold off, except for a few that are kept to service the females, for haulage, transport, and other such purposes [30]. Higher seroprevalence of camel brucellosis was recorded in adult animals (1.8%) compared with younger groups (0%). The result was not statistically significant (*p* > 0.5). The present finding was in agreement with previous reports where a higher seroprevalence of camel brucellosis was noted in adults (64.8%) than (35.2%) young animals in the southern province of Jordan [26]. Similarly, a higher seroprevalence (13.8%) in adults than (0%) in young camels in selected districts of the Afar region in Ethiopia [32]. Age has been referred to as one of the intrinsic factors associated with brucellosis in animals. Brucellosis is known as a disease of adult animals since susceptibility increases after sexual maturity and pregnancy [33]. This is due to the fact that, *Brucella spp*. Presents tropism to the reproductive tract due to the production of erythritol sugar in the fetal tissues [34]. Long-time contact with infected animals or with the environment also contributes to the higher prevalence of brucellosis in adult animals, which are significantly seen in those herds without culling of positive animals [23].

The high seroprevalence of camel brucellosis was recorded in the 6.8% of poor body conditioned animals compared with those with good body condition 1.03%. The difference in the seroprevalence of camel brucellosis in the two-body condition categories was found to be statistically significant (*p* < 0.05). This may be due to the body immunity level of the animals [35].

Higher seroprevalence of camel brucellosis was observed in large herd at 3.8% than medium and small herd sizes 1.7% and 1.01%, respectively. Similarly, same scenario of higher sero-prevalence of camel brucellosis 8.33%, 6.67% and 23.08% was recorded in small, medium and large herd sizes, respectively [24]. The reasons for this might be attributed to the easy contact among the animals favouring higher chances of bacterial transmission [36].

The sero-prevalence of camel brucellosis on the basis of abortion status was found to be 12.5% and 1.05% in aborted and nonaborted animals, respectively. The difference in the sero-prevalence of camel brucellosis between aborted and nonaborted groups was found to be statistically significant (*p* < 0.05). In a previous study, a statistically significant difference (*p* < 0.05) was revealed in camels with a previous history of abortion than nonaborted categories [37]. Similarly, among the reproductive disorders, abortion was significantly associated with sero-prevalence of bovine brucellosis from northern Ethiopia [38]. The association between *Brucella* infection and abortion in camels has been described in other studies [39] and is a well-recognized sign in most *Brucella* infections. The underlying cause of abortion in brucellosis is linked to its ability to adapt to the environmental conditions encountered in its intracellular replicative niche, including low levels of nutrients and oxygen, acidic pH, and reactive oxygen intermediates [40]. Inside the cells, *Brucella* has the ability to interfere with intracellular trafficking, preventing fusion of the *Brucella* containing microphages (phagosomes) with lysosome markers, and directing the vacuole toward a compartment that has rough endoplasmic reticulum (RER), which is highly permissive to intracellular replication of *Brucella* [41]. Then*, Brucella spp* disseminates throughout the body and induce suppression of the transcription of proinflammatory mediators in trophoblastic cells at very early stages of infection in females. After an initial suppression of proinflammatory transcripts, Brucella bacteria induce expression of proinflammatory chemokines, which finally results in abortion in female animals [41, 42].

The seroprevalence of camel brucellosis in terms of contact established with other ruminant animals revealed that animals in contact with small ruminants had higher seroprevalence (2.4%) compared with cattle (0%), and cattle and small ruminants (1.4%). The seroprevalence of camel brucellosis in animals kept alone was found to be even lower than 1%. However, there was no statistically significant difference (*p* > 0.05) in the seroprevalence of camel brucellosis and contact with other ruminant animals. Previous studies underscored the higher chance of bacterial transmission between camels and small ruminant with brucellosis [9, 43].

## 5. Conclusions

Brucellosis is a disease of high economic and public health importance and has a worldwide distribution. It is also widely spread in the camelproducing horns of African countries, including Ethiopia. The present study showed relatively lower seroprevalence of camel brucellosis in and around Dire Dawa, eastern Ethiopia. Despite the lower sero-prevalence of camel brucellosis, seropositivity was significantly associated with the previous history of abortion and body condition. Although relatively lower seroprevalence of camel brucellosis was noticed in the present study, the seropositive animals may serve as future foci of infection, pose a public health risk, and lead to low productivity and market value of camels. Further research intended for the isolation of causative agents and the identification of species and biotypes in Ethiopia should be conducted in camel rearing areas of the country. Camel pastoralists are often marginalized from public service facilities and information. Thus awareness on public health importance of camel brucellosis and its prevention is quite necessary.

## Figures and Tables

**Table 1:** Study population and sample size per district.

PAs selected	Camel population in the PA	Number of camel herds sampled	Animals sampled per PA	Number of respondents
Legebira	2900	7	63	30
BishanBehe	3500	11	72	30
Koriso	4150	14	95	30
Dujuma	5550	18	120	30
Total	17,100	50	350	120

**Table 2:** Overall seroprevalence of camel brucellosis using mRBPT and CFT tests.

Variables	Categories	No. Tested	mRBPT prevalence	CFT prevalence
Sex	Male	143	10 (7%)	2 (1.4%)
	Female	207	19 (9.2%)	5 (2.4%)
Age	≤3 yrs.	54	2 (3.7%)	0
	4–10 yrs.	167	10 (6%)	3 (1.8%)
	≥11 yrs.	129	17 (13.2%)	4 (3.1%)
Body condition	Good	291	22 (7.6%)	3 (1.03%)
	Poor	59	7 (11.9%)	4 (6.8%)
Herd size	Small	99	6 (6.1%)	1 (1.01%)
	Medium	172	13 (7.6%)	3 (1.7%)
	Large	79	10 (12.7%)	3 (3.8%)
Abortion status	Aborted	24	6 (25%)	3 (12.5%)
	Nonaborted	191	3 (1.6%)	2 (1.1%)
Contact with other ruminants	Single	196	21 (10.7%)	2 (1%)
	Cattle	42	1 (2.4%)	0
	Small ruminants	42	3 (7.1%)	1 (2.4%)
	Cattle and small ruminants	70	4 (5.7%)	1 (1.4%)

**Table 3:** Seroprevalence of camel brucellosis based on sex, age, and body condition using CFT as confirmatory.

Variables	Categories	No. Tested	Prevalence	*χ* ^2^	*P*-value
Sex	Male	143	2 (1.4%)	0.31	0.58
	Female	207	5 (2.4%)		
Age	<3 yrs	54	0 (0%)	1.25	0.536
	4–10 yrs	167	3 (1.8%)		
	≤11 yrs	129	4 (3.1%)		
Body condition	Good	291	3 (1.03%)	8.3	0.004^*∗*^
	Poor	59	4 (6.8%)		

^*∗*^Significantly different.

**Table 4:** Seroprevalence of camel brucellosis based on abortion status, herd sizes, and contact with other ruminants using CFT as confirmatory.

Variables	Categories	No. Tested	Prevalence	*χ* ^2^	*P*-value
Herd size	Small herd	99	1 (1.01%)	1.85	0.396
	Medium herd	172	3 (1.7%)		
	Large herd	79	3 (3.8%)		
Abortion status	Aborted	24	3 (12.5%)	12.3	0.000^*∗*^
	Nonaborted	191	2 (1.1%)		
Contact with other ruminants	Single	196	2 (1%)	0.17	0.982
	Cattle	42	0 (0%)		
	Small ruminants	42	1 (2.4%)		
	Cattle and small ruminants	70	1 (1.4%)		

^*∗*^Significantly different.

## Data Availability

Data used to support the findings of this study are available from the corresponding author upon request.
